# Case Report: Masseter hypertrophy: A case and review

**DOI:** 10.12688/f1000research.140341.1

**Published:** 2023-10-04

**Authors:** Prasanna R. Sonar, Aarati S. Panchbhai

**Affiliations:** 1 Department of Oral Medicine and Radiology, Sharad Pawar Dental College & Hospital, Datta Meghe Institute of Higher Education & Research, Wardha, Maharashtra, 442001, India

**Keywords:** computed tomography, magnetic resonance imaging, masseter muscle hypertrophy

## Abstract

Masseter muscle hypertrophy is a rare condition with no known etiology that has become more prevalent due to growing aesthetic concerns. It is distinguished by either unilaterally or bilaterally enlarged masseter muscles. Its origin is still unknown. This study discusses a clinically confirmed case of unilateral masseter muscle hypertrophy and provides an outline of the condition's etiology, prevalence, diagnosis, and course of treatment.

## Introduction

Anatomically, the masseter muscle is a robust, two-layered quadrate muscle. It originates from the inferior and deep surface of the zygomatic arch and mostly enters into the inferior lateral side of the mandibular ramus. ^1^ Unilateral or bilateral masseter muscle expansion is known as masseter hypertrophy. It may be asymptomatic or frequently accompanied by discomfort, which can occasionally be mistaken for parotid gland swelling. ^2^^–^^4^ For the functional, aesthetic, and differential diagnosis of head and neck pathology, it is an uncommon condition with an unknown cause. However, the most common reason patients with masseteric hypertrophy seek treatment is because of their facial appearance. ^5^ The case report's objectives are to outline the signs and symptoms of idiopathic masseter hypertrophy, offer a differential diagnosis, and recommend a course of action.

## Case report

**Figure 1.**
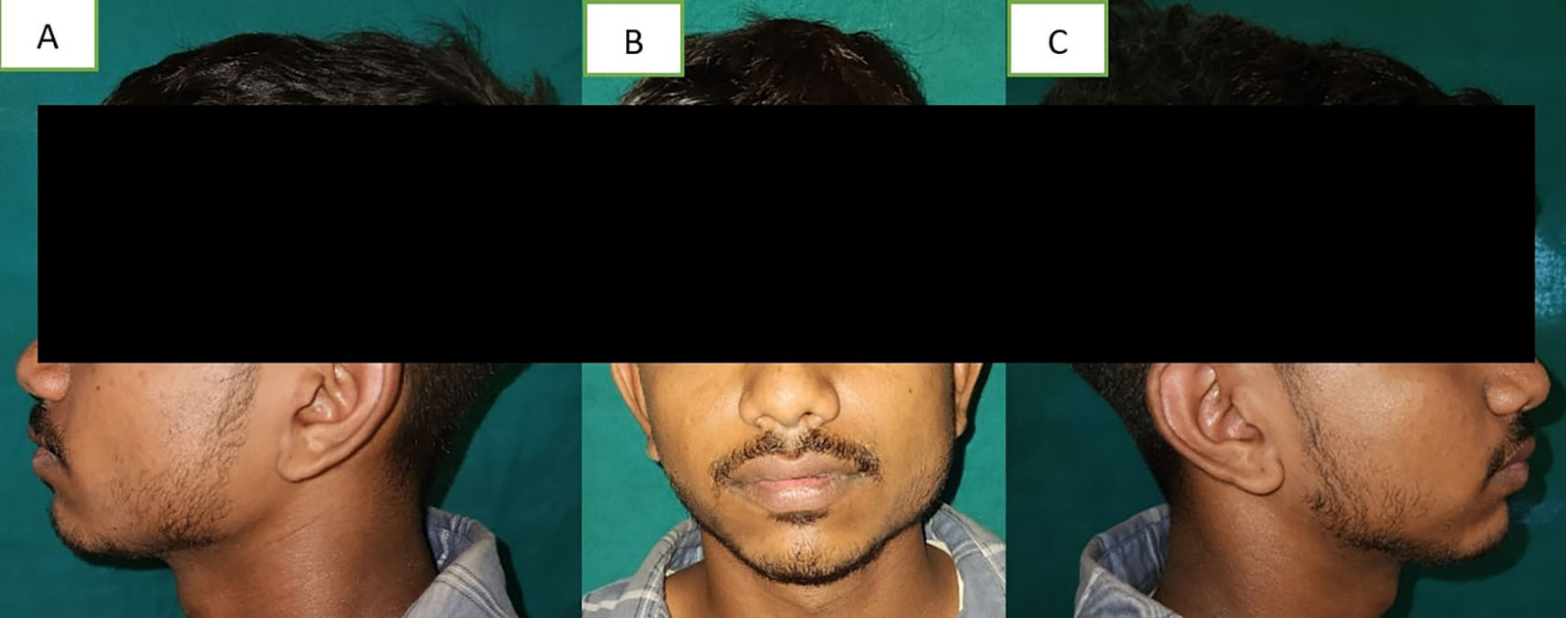
A – left profile, B – front profile, C – right profile. The masseter on the left side was broader than on the right side.

A 19-year-old male visited the dental hospital's oral medicine department. Due to unilateral facial swelling, the patient complained of an unattractive appearance ( Figure 1). The patient explained that the enlargement, which had been asymptomatic until now, had gradually increased since birth. Additionally, he had never before experienced trouble opening his mouth or temporomandibular symptoms. There was no family history of any such swelling, no history of face injuries, dental anomalies, or clicking in the temporo-mandibular joint.

**Figure 2.**
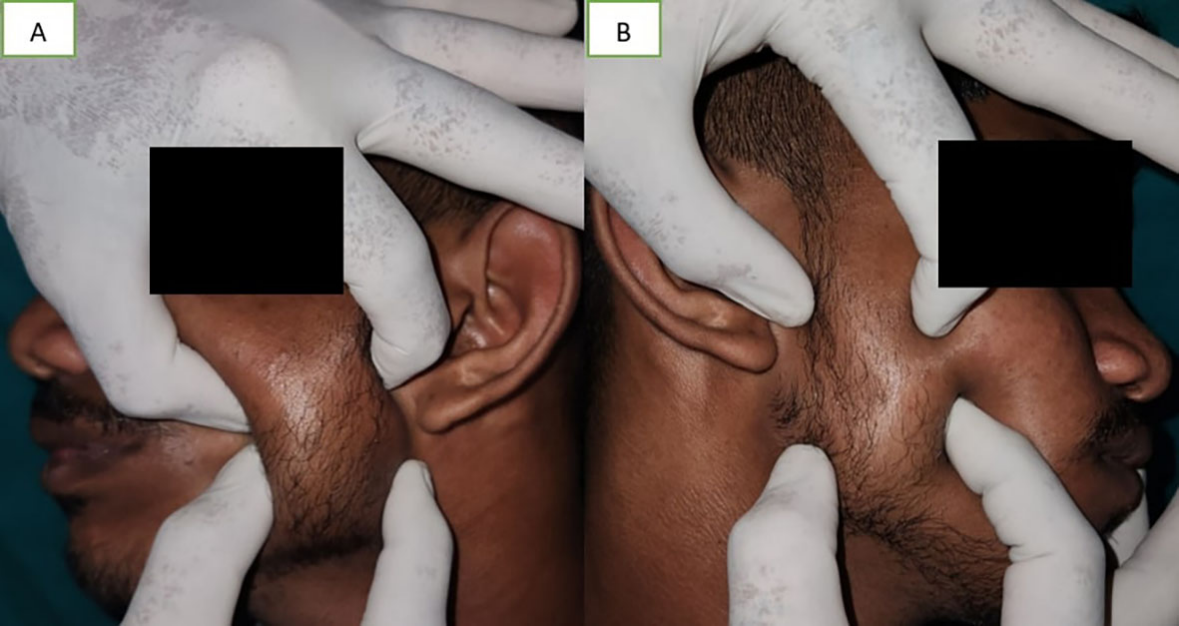
A – palpation of left masseter muscle, B – palpation of right masseter muscle. The masseter on the left side was broader than on the right side.

A soft to firm unilateral tissue mass was discovered during a clinical examination over the left body, close to the mandibular angle, which became prominent when the patient clenched their jaws ( Figure 2). When the left masseter was palpated, the inferior portion of the muscle showed increased size. The masseter on the left side was broader than on the right side. Due to the increase in muscle size and stress, there was compensatory hypertrophy at the insertion of muscle. Bone spur growth and the prominence of the mandibular angle were felt ( Figure 2).

The jaws moved normally as they opened and closed. During the occlusion, there was no midline deviation seen. Both an intra-oral and extra-oral examination revealed no abnormalities or possible etiological factors. There was no indication of a para-functional habit.

**Figure 3.**
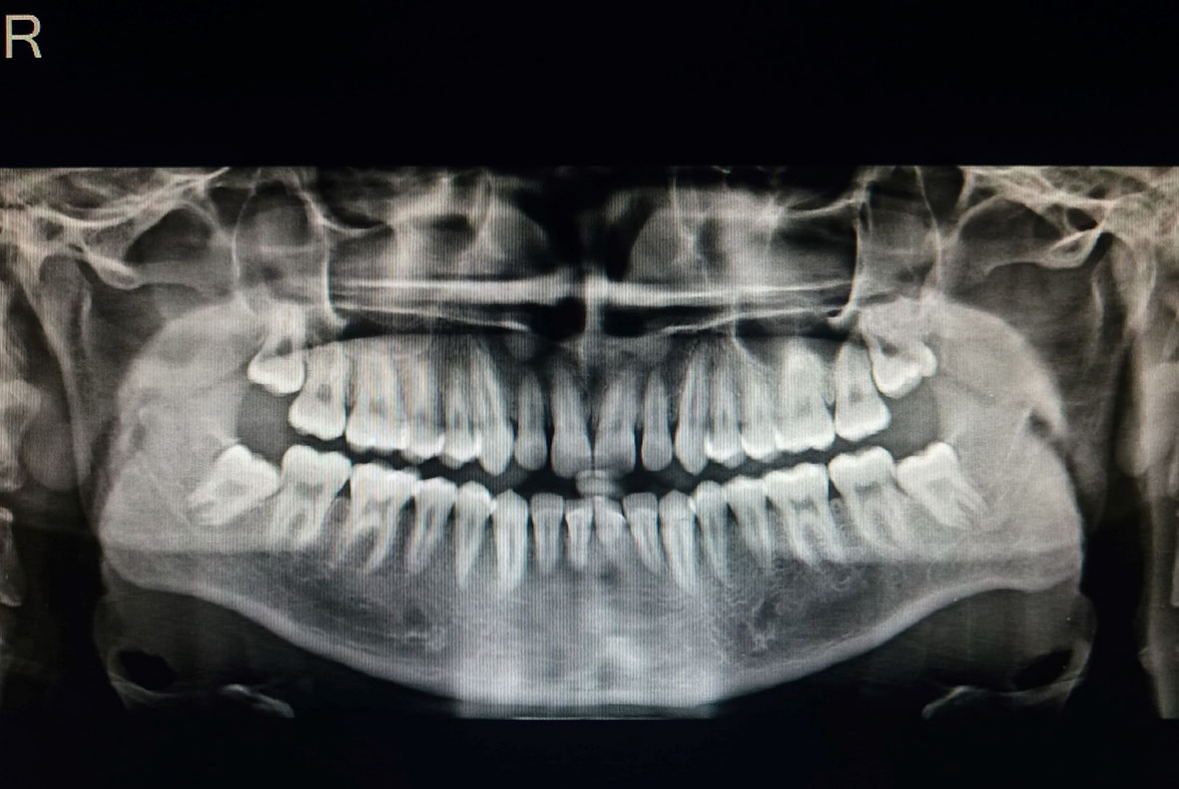
Orthopantomogram: the antagonial notch on left side was slightly more pronounced.

An orthopantomogram (OPG) was performed to look for any pathology ( Figure 3). Compared to the right side, the antagonial notch on the OPG scans was slightly more pronounced. Other anomalies were not found in OPG.

When we informed the patient about the masseter hypertrophy in this case, the patient declined further therapy. As a result, there was no treatment given in this asymptomatic patient.

## Discussion

### Prevalence

Asian Americans are more likely to be involved. ^6^^,^^7^ Without regard to gender, the second and third decades of life have the highest incidence rates. In their analysis of 108 instances, Baek *et al.* showed that the average patient age was 30 years old, 60% of them had bilateral involvement, and 57% of them were men. ^7^

### Etiology

According to some authors, the word “hypertrophy” may be deceptive because it refers to an increase in fiber count rather than cell size. ^8^

The cause of the majority of instances is unknown, despite the fact that numerous potential culprits, including malocclusion, bruxism, clenching, and abnormalities of the temporo-mandibular joint, have been proposed. ^4^^,^^8^

### Signs

In addition to being crucial for proper mastication, the masseter muscle is also crucial for facial aesthetics. Asymptomatic chronic expansion of one or both masseter muscles, known as masseteric hypertrophy, is typically the main complaint in cases of this condition.

The mandibular angle's bone spurs are frequently present observations. Bloem and Hoof asserted that this finding cannot be used as a diagnostic tool because 20% of healthy individuals have it. According to Guggenheim and Cohen, ^9^ bone spurs are brought on by periostal irritation and new bone formation in response to the muscles bundles' elevated pressures.

### Differential diagnosis

It is important to correctly diagnose idiopathic masseter muscle hypertrophy because it can be mistaken for other conditions. Among these are masseter tumor, salivary gland disease, parotid tumor, parotid inflammatory disease, and intrinsic masseter muscle myopathy (caused by hypotrophy or hypoplasia on the contralateral side). ^10^^-^^12^

A sialography is necessary to rule out this option since the correct diagnosis is more challenging in unilateral cases and necessitates a differential diagnosis with parotid gland abnormalities. ^13^^,^^14^

### Diagnosis

Clinical examination, medical history, imaging modalities' findings, and muscle palpation can all be used to diagnose masseter hypertrophy. Visual inspection and palpation revealed a consistent muscle mass in masseteric hypertrophy, and the patient's forced bite could be felt to compress the muscle. Other benign and malignant neoplasms, on the other hand, are characterized by irregular and nodular development. The size and position of bucco-masseteric masses can be determined by sonography, computed tomography (CT), and magnetic resonance imaging (MRI). ^5^ For the examination of hyperostosis at the point of muscle attachment in benign masseteric hypertrophy, CT scanning is essential. ^5^ The panoramic radiograph is the most feasible diagnostic to support the clinical diagnosis when the physical examination points to masseteric hypertrophy. ^5^ Ultrasonography of the region confirmed the uniform increased muscle mass with characteristics of normal musculature without any cystic, nodular or irregular foci. ^5^

The use of CT is a well-established technique for delivering a wealth of details about nearby buildings and notable landmarks. Due to its high-quality imaging of bone structures and direct bone imaging, which is not achievable with MRI since cortical bone produces no detectable signal, CT scanning is essential in cases with masseter muscle hypertrophy with bone flaring. However, in this case report, the hypertrophied area and the border between the medial and lateral pterygoid could not be limited by CT. However, because muscle structural signals from the damaged side of the body are stronger than those from the unaffected side, MRI made it easier to diagnose the condition. ^9^

### Management

There have been several reported treatments for masseter hypertrophy, ranging from non-invasive medical procedures to invasive surgical procedures. Some examples of conservative treatment include occlusal correction, relaxation therapy, botulinum therapy, spasmolytics, tranquilizers, and antidepressant therapy. Compared to surgical treatments, conservative techniques have both benefits and drawbacks. ^15^^,^^16^ Despite being an intrusive treatment, surgical therapy is nevertheless favored for more dependable and long-lasting results. Masseteric enlargement typically doesn't require treatment. Reassurance, tranquilizers or muscle relaxants, psychiatric care, and injection of extremely modest dosages of botulinum toxin type A are examples of non-surgical therapeutic techniques. ^17^ To fix malocclusions and premature contacts, dental restorations and occlusal modifications are crucial. Habits that are dysfunctional must be avoided. Patients may choose to have cosmetic surgery in this situation to lessen the prominence of the mandibular angle bone. ^18^

A benign ailment called masseter muscle hypertrophy normally doesn't require surgery.

Due to improper diagnosis in these circumstances, needless biopsies, exploratory surgeries, and even radiotherapy for parotid tumors may be performed. To rule out other disorders, conventional radiography examinations, CT scans, and MRI scans are useful. ^19^

## Conclusion

Masseter hypertrophy can have a substantial impact on the aesthetics and functionality of the face, changing the jaw's appearance and function. To choose the best course of treatment, it is crucial to precisely evaluate the underlying problem.

### Case report consent

Written informed consent for publication of their clinical details and clinical images was obtained from the patient.

## Data Availability

All data underlying the results are available as part of the article and no additional source data are required. Zenodo: CARE checklist for ‘Case Report: Masseter hypertrophy: A case and review’. https://doi.org/10.5281/zenodo.8307408. Data are available under the terms of the Creative Commons Attribution 4.0 International license (CC-BY 4.0).
