# Supplementary material for: Case Report: Masseter hypertrophy: A case and review
Source: F1000Res. 2024 Jul 3;12:1263. Originally published 2023 Oct 4. [Version 2] doi: 10.12688/f1000research.140341.2 (PMC11258545; doi:10.12688/f1000research.140341.2)
Supplement: Peer review history

# Reviewer report: Reviewer response for version 2

**DOI:** 10.5256/f1000research.168309.r298879

**Published:** 2024-07-18

**Authors:** Vini Mehta

**Affiliations:** 1 Dr. D.Y. Patil Dental College and Hospital, Pune, India

It can be indexed. All comments are satisfactorily addressed.

Are enough details provided of any physical examination and diagnostic tests, treatment given and outcomes?

Yes

Is the case presented with sufficient detail to be useful for other practitioners?

No

Is sufficient discussion included of the importance of the findings and their relevance to future understanding of disease processes, diagnosis or treatment?

No

Is the background of the case’s history and progression described in sufficient detail?

No

Reviewer Expertise:

Public health

I confirm that I have read this submission and believe that I have an appropriate level of expertise to confirm that it is of an acceptable scientific standard.

# Reviewer report: Reviewer response for version 1

**DOI:** 10.5256/f1000research.153683.r265347

**Published:** 2024-05-16

**Authors:** Vini Mehta

**Affiliations:** 1 Dr. D.Y. Patil Dental College and Hospital, Pune, India

1. Please elaborate the introduction and discussion. 
2. Remove sub headings from the discussion.
3. Is a review of the literature included with your case? 
4. The references used in the case report are very old. Kindly update the references and ensure that 70% of the references are used from last 5 years. 

Are enough details provided of any physical examination and diagnostic tests, treatment given and outcomes?

Yes

Is the case presented with sufficient detail to be useful for other practitioners?

No

Is sufficient discussion included of the importance of the findings and their relevance to future understanding of disease processes, diagnosis or treatment?

No

Is the background of the case’s history and progression described in sufficient detail?

No

Reviewer Expertise:

Public health

I confirm that I have read this submission and believe that I have an appropriate level of expertise to confirm that it is of an acceptable scientific standard, however I have significant reservations, as outlined above.

# Reviewer report: Reviewer response for version 1

**DOI:** 10.5256/f1000research.153683.r246352

**Published:** 2024-02-24

**Authors:** Nabeel Ayappali Kalluvalappil

**Affiliations:** 1 Oral Medicine and Maxillofacial Radiology, College of Dentistry, University of Science and Technology of Fujairah, Fujairah, United Arab Emirates

Summary:

 The article provides a detailed examination of unilateral masseter muscle hypertrophy, presenting a case report of a 19-year-old male with unilateral facial swelling attributed to this condition. The patient's history, clinical examination findings, diagnostic tests, treatment options, and outcomes are thoroughly discussed. Additionally, the article offers insights into the prevalence, etiology, differential diagnosis, and management of masseter muscle hypertrophy, contributing to the understanding and treatment of this rare condition.

 Suggestions: 

 1. To facilitate better understanding for the readers, it would be helpful to present the imaging findings more clearly.

Are enough details provided of any physical examination and diagnostic tests, treatment given and outcomes?

Yes

Is the case presented with sufficient detail to be useful for other practitioners?

Yes

Is sufficient discussion included of the importance of the findings and their relevance to future understanding of disease processes, diagnosis or treatment?

Yes

Is the background of the case’s history and progression described in sufficient detail?

Yes

Reviewer Expertise:

Oral Medicine, Maxillofacial Radiology, Dental Radiology, Head and Neck Ultrasonography, CBCT, Forensic Odontology, Red and White Oral lesions, Intentional Replantation, Modern technologies in Dentistry, Lasers,  Implants, Oral cancer, Precancerous lesions and conditions

I confirm that I have read this submission and believe that I have an appropriate level of expertise to confirm that it is of an acceptable scientific standard.

# Response

**Published:** 2024-05-02

**Authors:** Prasanna Sonar

**Affiliations:** Oral Medicine and Radiology, Datta Meghe Institute of Higher Education and Research, Wardha, Maharashtra, India

Respected Sir/ Maam,

 Photos of imaging modalities are not available.

 Kindly consider.

 thankyou.
